# Genomic data support management of anadromous Arctic Char fisheries in Nunavik by highlighting neutral and putatively adaptive genetic variation

**DOI:** 10.1111/eva.13248

**Published:** 2021-05-27

**Authors:** Xavier Dallaire, Éric Normandeau, Julien Mainguy, Jean‐Éric Tremblay, Louis Bernatchez, Jean‐Sébastien Moore

**Affiliations:** ^1^ Institut de Biologie Intégrative et des Systèmes (IBIS) Université Laval Québec QC Canada; ^2^ Centre d’Études Nordiques (CEN) Université Laval Québec QC Canada; ^3^ Département de Biologie, Université Laval Québec QC Canada; ^4^ Ministère des Forêts, de la Faune et des Parcs Québec QC Canada

**Keywords:** anadromous salmonid, Arctic, local adaptation, marine ecosystems, population genomics

## Abstract

Distinguishing neutral and adaptive genetic variation is one of the main challenges in investigating processes shaping population structure in the wild, and landscape genomics can help identify signatures of adaptation to contrasting environments. Arctic Char (*Salvelinus alpinus*) is an anadromous salmonid and the most harvested fish species by Inuit people, including in Nunavik (Québec, Canada), one of the most recently deglaciated regions in the world. Unlike many other anadromous salmonids, Arctic Char occupy coastal habitats near their natal rivers during their short marine phase restricted to the summer ice‐free period. Our main objective was to document putatively neutral and adaptive genomic variation in anadromous Arctic Char populations from Nunavik and bordering regions to inform local fisheries management. We used genotyping by sequencing (GBS) to genotype 18,112 filtered single nucleotide polymorphisms (SNP) in 650 individuals from 23 sampling locations along >2000 km of coastline. Our results reveal a hierarchical genetic structure, whereby neighboring hydrographic systems harbor distinct populations grouped by major oceanographic basins: Hudson Bay, Hudson Strait, Ungava Bay, and Labrador Sea. We found genetic diversity and differentiation to be consistent both with the expected postglacial recolonization history and with patterns of isolation‐by‐distance reflecting contemporary gene flow. Results from three gene–environment association methods supported the hypothesis of local adaptation to both freshwater and marine environments (strongest associations with sea surface and air temperatures during summer and salinity). Our results support a fisheries management strategy at a regional scale, and other implications for hatchery projects and adaptation to climate change are discussed.

## 1 INTRODUCTION

Intraspecific diversity is an important part of biodiversity, especially at higher latitudes where species richness is relatively low (Pamilo & Savolainen, 1999). Throughout the Palearctic, the periodic range contractions and expansions brought about by glacial cycles have shaped this diversity through bottlenecks and genetic drift (Hewitt, 2000) and particularly so in fishes (April et al., 2013; Bernatchez & Wilson, 1998). Local adaptation can also arise when species experience different environmental conditions over their geographic ranges (Kawecki & Ebert, 2004; Williams, 1966). Local adaptation has been studied extensively via reciprocal transplant and common‐garden field experiments, but these approaches do not provide information on the molecular basis of adaptation (Tiffin & Ross‐Ibarra, 2014). New genomic methods are now commonly used to advance our understanding of local adaptation (Grummer et al., 2019; Luikart et al., 2018). Such adaptive genomic variation, as well as contemporary population genetic structure, are of great interest for both conservation and management to ensure actions target biologically significant units (Bernatchez et al., 2017; Funk et al., 2012).

Salmonids are a diverse family of fishes with high economic and cultural importance. Anadromous salmonids are philopatric, that is, returning to their natal habitat for spawning (Quinn, 1993) and this behavior is known to reduce gene flow amongst populations, thus promoting genetic differentiation and local adaptation at fine spatial scales (reviewed in Fraser et al., 2011). The Arctic Char (*Salvelinus alpinus*) is a salmonid fish with a circumpolar distribution and is known for its great diversity in life‐history characteristics (Klemetsen, 2010). Anadromous individuals spend 3–9 years in cold oligotrophic freshwater at birth (Johnson, 1980), then complete annual migrations between marine habitats for summer foraging and lakes for overwintering. Although straying (i.e., an upstream migration in a non‐natal river system) can occur, several studies have shown that Arctic Char maintained philopatric behavior during reproductive years, limiting effective dispersal (Gyselman, 1994; Moore et al., 2013, 2017). Moore et al. (2013) also argued that gene flow could be sufficiently low to allow for local adaptation among populations of eastern Baffin Island in the Canadian Arctic, while Moore et al. (2017) provided some genomic evidence for local adaptation to natal rivers at a fine spatial scale. In marine environments, Arctic Char tend to stay near the surface (<3 m), with occasional dives up to 50 m (Harris et al., 2020; Spares et al., 2012), and, unlike other salmonid species, preferably use near‐shore habitats within approximatively 100 km from their natal river's mouth (Dempson & Kristofferson, 1987; Layton et al., 2020; Moore et al., 2016).

Potentially strong demographic bottlenecks during glaciations have greatly reduced intraspecific genetic diversity and lead to divergent glacial lineages that survived in different refugia (Bernatchez & Wilson, 1998; Hewitt, 2000). Secondary contact between lineages has been shown to have commonly occurred among temperate freshwater fishes (e.g., Bradbury et al., 2015; Rougeux et al., 2019; Turgeon & Bernatchez, 2001). In North America, Arctic Char comprises four known mitochondrial DNA (mtDNA) lineages associated with distinct glacial refugia (Brunner et al., 2001; Moore et al., 2015). The Arctic lineage is predominant in the south‐eastern Arctic (Brunner et al., 2001; Moore et al., 2015), but populations across northern Labrador are admixed with the Atlantic lineage (Salisbury et al., 2019).

**FIGURE 1:**
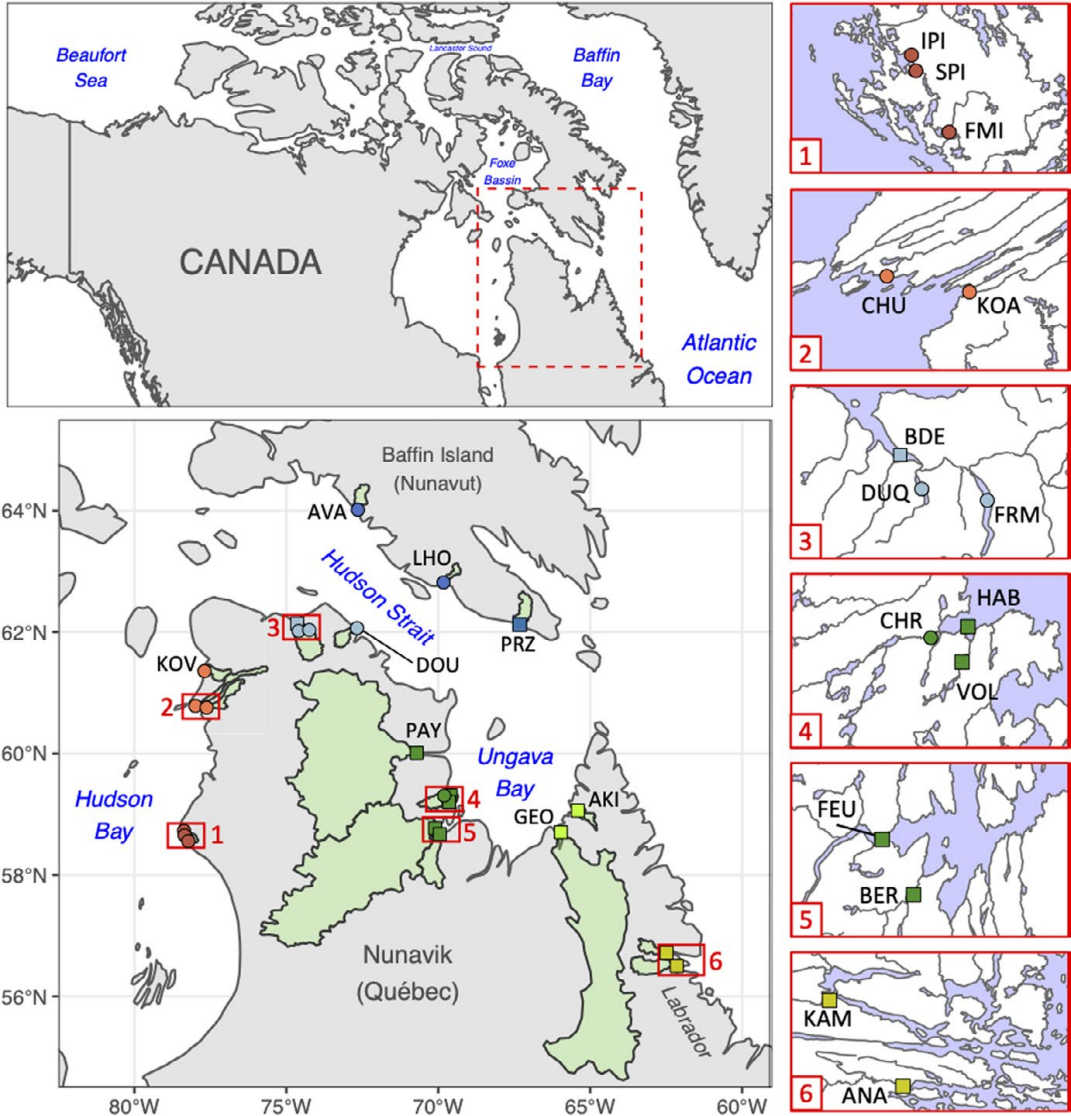
Sampling locations in Nunavik (Québec, Canada) and bordering regions. Red extents numbered 1–6 are magnified to show neighboring sampling locations. Samples were either used for genotyping by sequencing (GBS) only (round) or for GBS and mitochondrial DNA sequencing (square). Catchment area for each site is displayed in green

Nunavik, situated in northern Québec (Canada), is one of the last regions in North America to have deglaciated following the last glacial maximum (LGM; Dalton et al., 2020; Dyke, 2004). It is bordered by the Hudson Bay, Hudson Strait, and Ungava Bay (Figure 1). These three marine regions are contrasted in their surface temperature, salinity, productivity, and tidal regimes (Prisenberg, 1984; Savard et al., 2014). In remote coastal communities of Nunavik, Arctic Char subsistence fisheries are key to food security (Laflamme, 2014) and rapid demographic growth has increased harvesting pressures on wild fish populations (Martin, 2011). The current study applies population genomic methods to investigate the relative role of neutral vs. adaptive processes in shaping contemporary population structure of anadromous Arctic Char over 2000 km of coastline in Nunavik. This will provide a genomic context of relevance for sustainable fisheries management.

Since we expected the contemporary genetic structure of Arctic Char populations in Nunavik to be heavily influenced by their recent postglacial recolonization history, we first sequenced the mitochondrial control region to (i) test for the presence of the Atlantic lineage in Nunavik, placing the present study in the context of previous phylogeographic work in the region that relied heavily on mtDNA. We then used genotyping by sequencing (GBS) to determine whether Arctic Char populations from different regions of Nunavik (ii) are genetically differentiated, (iii) differ in their genetic diversity, and (iv) follow patterns of isolation‐by‐distance. Additionally, as migration in Arctic Char has been found to be predominantly coastal (Moore, 1975; Moore et al., 2013, 2015; Spares et al., 2012), we tested whether (v) the Hudson Strait, a 120‐km‐wide open water area, acts as a barrier to gene flow between populations on opposite shores. Finally, since we expected broad‐scale variation in environments to be the source of divergent selective pressures, we attempt to (vi) detect evidence of local adaptation to both freshwater and marine environments around Nunavik and bordering regions.

## 2 METHODS

### 2.1 Sampling and DNA extraction

Arctic Chars were sampled in 23 water bodies across Nunavik, southern Baffin Island, and Labrador (Figure 1). In Nunavik and Baffin Island, adult fish were harvested during their upstream migration using gillnets or counting weirs. In most localities throughout Nunavik, sampling locations were selected in concert with local and regional Inuit wildlife managers, and sampling was done with the assistance of local Inuit guides to prioritize fish populations with an importance for traditional fishing. In two locations (Deception Bay and Hopes Advance Bay), samples were taken in the estuary as well as in two tributary rivers. Juvenile fish were captured by electrofishing in two rivers near Nain, Labrador. Adipose fin clips were collected from each fish and preserved in ethanol 95%. DNA was extracted from fin clips using a modified version of Aljanabi and Martinez (1997). Agarose gel electrophoresis was used to assess DNA quality, and the quantity and quality of DNA were evaluated by NanoDrop spectrophotometer (Thermo Scientific).

### 2.2 Mitochondrial DNA analysis

To assess whether the Arctic and Atlantic glacial lineages hybridized in Nunavik, we sequenced mtDNA for a subset of individuals from sampling sites in the eastern part of our study area (*n* = 10–34 per site). The entire mitochondrial control region was amplified with primers Tpro2 (Brunner et al., 2001) and SalpcrR (Power et al., 2009) and we sequenced 499 bp of the control region's left domain according to methods outlined in Power et al. (2009). Control region sequences were aligned on the reference haplotype set described in Salisbury et al. (2019) using the Geneious (6.1.8; www.geneious.com) alignment tool with parameters set to default and the closest matching haplotype was reported.

### 2.3 GBS library preparation, sequencing, and single nucleotide polymorphisms (SNP) calling

Ten (10) µl of DNA was normalized to a concentration of 10 ng/µl using Quant‐iT Picogreen dsDNA Assay Kit (Invitrogen) for precision quantification. GBS libraries were prepared with a modified version of the Abed et al. (2019) two‐enzyme GBS protocol, using *Pst*I and *Msp*I restriction enzymes. Samples were randomly assigned to libraries to limit batch effects. Sequencing was performed at the Plateforme d’Analyses Génomiques (PAG) at IBIS, Université Laval (http://www.ibis.ulaval.ca/) on Ion Torrent sequencers with p1v3 chips and a median target of 80 million single‐end reads per chip. Each library was sequenced on three separate chips for each batch of 96 individuals, and the volume of DNA from each sample was adjusted after the first chip to reduce the unbalanced representation of individuals in sequences.

We processed the data and filtered the SNP dataset using a RADseq workflow (https://github.com/enormandeau/stacks_workflow) built around STACKS 2.5 (Rochette et al., 2019). Briefly, the sequences were trimmed at 80 bp and aligned on a *Salvelinus* sp. reference genome (ASM291031v2; NCBI RefSeq: GCF_002910315.2; Christensen et al., 2021) using bwa mem (‐k 19 ‐c 500 ‐O 0,0 ‐E 2,2 ‐T 0) in BWA‐0.717 (r1188; Li & Durbin, 2009) and samtools view (‐Sb ‐q 1 ‐F 4 ‐F 256 ‐F 2048) in samtools 1.8 (Li et al., 2009). SNPs were called on polymorphic genotypes with at least 4X coverage per sample (‐m), present in at least 60% of samples of each sampling sites (‐r) and with the minor allele present in a minimum of three samples. This minor allele sample (MAS) filter is akin to minor allele frequency (MAF), but unlike MAF it is not artificially boosted by frequent RADseq genotyping errors (Linck & Battey, 2019). Samples with more than 20% of missing data or with heterozygosity (*F*_IS_) under −0.2 were removed. For SNP calling and quality control, we combined our samples with 119 other samples from three sampling sites that were not included in our final dataset, as they either had too few samples, had no access to sea, or targeted a population reintroduced from a hatchery brood.

Salmonid fishes have a common ancestor that experienced a whole‐genome duplication approximately 60 MYA (Crête‐Lafrenière et al., 2012), and many genetic markers identified in our analyses are expected to be situated on paralogous loci of similar sequences. While these loci may be important for adaptation (Kondrashov, 2012), they were removed due to the fact that these markers do not behave like biallelic SNPs and because genotyping is difficult without very high coverage (>100 reads; Dufresne et al., 2014). SNPs on duplicated loci were categorized and filtered using an adapted *HDplot* procedure (McKinney et al., 2017), which identifies paralogs by visually comparing the allelic ratio, the proportion of heterozygotes and homozygotes of the rare allele, and the *F*_IS_ value for each SNP.

We also filtered the markers to avoid physically linked SNPs while keeping a maximum of information: For each pair of SNPs within 50,000 bp, we assessed linkage considering samples without missing data where at least one of the two genotypes contains the rare variant. If the two markers had identical genotypes in more than 50% of these samples, the pair was considered linked and only the first SNP was kept. To mitigate the risk of our inference regarding population structure being affected by an uncharacterized sex‐ratio bias (Benestan et al., 2017), we identified sex‐related markers by performing a redundancy analysis (RDA) using individual genotypes from samples for which information on sex was available in Hudson Bay (*n* = 69) and Ungava Bay (*n* = 85). We classified SNPs as showing a statistically supported association with sex when they loaded with more than 2.5 standard deviation from the mean (*p* = 0.012) and removed those markers from the dataset. Finally, we measured pairwise relatedness (Yang et al., 2010) in vcftools v0.1.13 (Danecek et al., 2011) and eliminated five related individuals in separate sampling sites, as their high relatedness values are thought to be the result of technical artifacts.

### 2.4 Identification of putative neutral markers

Markers potentially under selection were identified using two methods: (1) pcadapt (Luu et al., 2017) and (2) BayeScan (Foll & Gaggiotti, 2008). SNPs identified as outliers by at least one method were removed to produce a neutral dataset. The R package *pcadapt* was used to identify outlier SNPs in relation to population structure according to a principal component analysis (PCA). The first 13 PCs were used, based on visual evaluation of PCA scores and scree plots, and SNPs with minor allele frequencies under 0.05 were excluded from the analysis. We then used BayeScan with prior odds of 1000 and other parameters set to default. For both tests, SNPs with a false detection rate (*q*‐value) under 0.05 were considered putatively under selection.

### 2.5 Basic statistics and population structure

Population genetic statistics were computed from the neutral dataset. Observed and expected heterozygosity was calculated by population using GenoDive v3.0 (Meirmans & Van Tienderen, 2004). We used vcftools v0.1.13 to measure the proportion of heterozygous SNPs for each individual, and we calculated the number of polymorphic SNPs in each population. Effective population sizes (N_e_) and 95% C. I. were estimated with Neestimator v2.01 (Do et al., 2014) using the linkage disequilibrium method on markers with minor allele frequencies over 0.05.

We used a principal coordinate analysis (PCoA) in the R package *dartR* (Gruber et al., 2018) to document population structure using again the neutral data set. We also estimated ancestry with the maximum‐likelihood approach implemented in ADMIXTURE (Alexander et al., 2009) with the number of genetic clusters (*K*) ranging from 1 to 20. We considered the value of *K* yielding the lowest cross‐validation error to be the most likely number of genetic groups. Based on this *K* value, we identified contiguous sampling sites where most individuals shared a common cluster membership and repeated the ADMIXTURE analysis within those sites with *K* ranging from 1 to 6. Considering that adult Arctic Char is expected to stray to nearby rivers, we regrouped sampling sites in connecting rivers and estuaries for the following analyses on genetic diversity and isolation‐by‐distance, with the exception of cases where ADMIXTURE showed strong population structure.

Spatial patterns of genetic diversity were compared between a priori regions, using the individual proportion of heterozygous SNPs. A nested ANOVA was performed with the regions as groups and the sampling sites as subgroups. To account for our unbalanced sampling design, we did a comparison of estimated marginal means (or least‐square means) on a linear mixed‐effect model with a random effect of the sampling site, in the R packages *glmmTMB* (Magnusson et al., 2017) and *emmeans* (Lenth, 2021).

### 2.6 Landscape genomics

Pairwise population *F*_ST_ were calculated with the R package *StAMPP* (Pembleton et al., 2013), and 1000 bootstraps were performed to estimate their significance value as the proportion of bootstrapped *F*_ST_ values under zero. *F*_ST_ calculations were performed on both the neutral and putatively adaptive datasets. The geographic marine distance separating sampling sites was measured between the coordinates at the mouth of sampled rivers by a least‐cost path in the R package *marmap* (Pante & Simon‐Bouhet, 2013) using NOAA bathymetric data at a 4‐minute resolution to discriminate land and sea.

We tested for the presence of isolation‐by‐distance (IBD) with a linear mixed‐effect model. Linearized *F*_ST_ (*F*_ST_/(1 – *F*_ST_); Slatkin, 1995), based on the neutral dataset, was used as the dependent variable and marine distance as a fixed effect. Patterns of IBD between pairs of sampling sites on the same coast versus pairs on either side of Hudson Strait were compared by the inclusion of a categorical fixed effect. To characterize the impact of Hudson Strait as a barrier to gene flow at different spatial scales, analyses were performed on all nonestuarine sites, then only on nonestuarine sites within 250 km of the Hudson Strait. To account for the nonindependence of pairwise distances, the model was run with a maximum‐likelihood population effect parameterization (MLPE) (Clarke et al., 2002) with and without restricted maximum likelihood (REML), using the MLPE.lmm function in the R package *ResistanceGA* (Peterman, 2018). Models without REML were compared with conditional Akaike information criterion (cAIC; Vaida & Blanchard, 2005) in the R package *cAIC4* (Säefken et al., 2019) and with marginal *R* ^2^ (Nakagawa & Schielzeth, 2013) in the R package *MuMIn* (Barton, 2019) for models with REML.

### 2.7 Gene–environment association

We used the ArcGIS software v10.4 (ESRI, 2011) to extract environmental data from BIO‐Oracle v2.0 (Assis et al., 2018), Marspec (Sbrocco & Barber, 2013), and WorldClim v2.0 (Fick & Hijmans, 2017) (Table 1, see Table S5 for values at sites). Tide data were obtained from FES2014, produced by Noveltis, Legos, and CLS and distributed by Aviso+, with support from Cnes (https://www.aviso.altimetry.fr/). Marine variables represent sea‐surface values for factors of potential biological importance for Arctic Char and were aggregated in a 20 km radius around each river mouth and within 5 km from the coast, as to best represent the local coastal environment based on existing knowledge of Arctic Char marine habitat use from other geographical regions (Moore et al., 2016; Spares et al., 2015). Freshwater variables comprise the area of the watershed upstream of the sampling site, as well as air temperature and precipitation statistics on these areas. Air temperature is commonly used as a proxy for freshwater temperature in remote areas, as supported by studies linking the growth rate of Lake Trout to air temperature (Black et al., 2013; Torvinen, 2017). To deal with multicolinearity, a PCA was performed on the scaled environmental factors, and the PCs with eigenvalues over 1 were used as explanatory variables in gene–environment association (GEA) analyses.

**TABLE 1:** Value range and source of environmental factors considered in gene–environment associations. PCA axis mostly associated with variables is indicated with sign (positive or negative) of the correlation in parentheses

Variable	Description	Value range	Unit	Temporal range	Database	Source	PCA axis
Marine variables							
Productivity	Annual mean primary productivity (carbon)	1.2 – 12.0	g m^−3^ day^−1^	2000–2014	Bio‐ORACLE	PISCES	PC1 (+)
O2	Annual mean dissolved molecular oxygen	327 – 364	mmol m^−3^	2000–2014	Bio‐ORACLE	PISCES	PC1 (‐)
SST_summer	Mean sea‐surface temperature (July to September)	0.5 – 6.8	°C	2002–2010	Marspec	WOA09	PC2 (+)
Turbidity	Annual mean diffuse attenuation	0.8 – 4.8	m^−1^	2002–2009	Bio‐ORACLE	Aqua‐MODIS	PC2 (+)
Salinity	Annual mean sea‐surface salinity	21.0 – 31.8	PSS	2000–2014	Bio‐ORACLE	ARMOR	PC2 (‐)
Tide_M2	Amplitude of M2 tidal constituent	17.5 – 372.6	—	—	FES2014	AVISO+	PC3 (+)
Freshwater variables							
P_winter	Mean precipitation of the coldest quarter	40.2 – 171.5	mm	1970–2000	WorldClim	GHCN	PC1 (+)
P_summer	Mean precipitation of the warmest quarter	114.7 – 250.1	mm	1970–2000	WorldClim	GHCN	PC1 (+)
T_winter	Mean air temperature of the coldest quarter	−25.0 – −19.7	°C	1970–2000	WorldClim	MODIS	PC1 (+)
T_min	Minimum air temperature of the coldest month	−29.0 – −26.3	°C	1970–2000	WorldClim	MODIS	PC1 (+)
T_summer	Mean air temperature of the warmest quarter	4.9 – 10.7	°C	1970–2000	WorldClim	MODIS	PC2 (+)
T_max	Maximum air temperature of the warmest month	9.3 – 16.4	°C	1970–2000	WorldClim	MODIS	PC2 (+)
WS_AREA	Upstream catchment area (watershed, log‐transformed)	30 – 43,496	km^2^	—	NHN	—	PC3 (+)

Abbreviations: ARMOR, Global Observed Ocean Physics Reprocessing; AVISO+, Archiving, Validation and Interpretation of Satellite Oceanographic data; GHCN, Global Historic Climate Network; MODIS, Moderate Resolution Imaging Spectroradiometer; NHN, National Hydrographic Network; ORAP, Global Ocean Physics Reanalysis ECMWF; PISCES, Global Ocean Biogeochemistry Non‐assimilative Hindcast; WOA09, World Oceanographic Atlas 2009.

Candidate SNPs associated with environmental variables were identified using the complete genomic dataset with a combination of two redundancy analyses (RDA) and latent factor mixed models (LFMM). Genes within 10,000 base pairs were recorded using bedtools (Quinlan, 2014) for candidate SNPs identified by at least two methods. We consulted the UniProt database (Uniprot Consortium, 2019) for information on biological gene functions in Atlantic salmon (*Salmo salar*), zebrafish (*Danio rerio*), or other organisms.

#### 2.7.1 RDA

We used RDA (Legendre & Gallagher, 2001) implemented in the R package *vegan* (Oksanen, 2012) to investigate multivariate correlations of genotypes in the form of allele frequencies by population with environmental variables. We took into account the structure in the data which derives from spatial patterns with a distance‐based Moran's eigenvector map (dbMEM; Borcard & Legendre, 2002; Peres‐Neot & Legendre, 2010). To build this map, we transformed pairwise marine distances measured in the section above in Euclidian distances by creating a Delaunay graph with the function chooseCN in the R package *adegenet* (Jombart, 2008). We then used the R package *adespatial* (Dray et al., 2020) to compute the dbMEMs from the Euclidian distances. This approach was chosen over mere longitude and latitude information to avoid the underestimation of marine migration distances, that is, by preventing considering for instance impossible movements through landmass. Eigenvectors reflecting positive spatial autocorrelation were used as covariables in a partial RDA. As spatial structure could potentially hide evolutionary significant environmental gradients, we performed a second RDA excluding spatial eigenvectors correlated to environmental factors, as suggested by Forester et al. (2018).

Spatial factors were first submitted to a backward model selection procedure with the function ordistep in *vegan*, and only statistically supported covariables at α = 0.05 were kept. We then repeated this model selection method for environmental factors for both RDAs. To identify SNPs associated with environmental components, *Z*‐scores were obtained for the distribution of individual SNP loadings on RDA axes explaining a significant portion of genetic variation (*p* < 0.05), and SNPs were defined as outliers if their maximal absolute *Z*‐score was over 3.5 (*p* < 0.0002).

#### 2.7.2 LFMM

The LFMM (Frichot et al., 2013) identifies loci–environment associations using a Bayesian mixed model with environmental variables included as fixed effects. Latent factors are derived from a PCA and used as random effects to control for population structure. Missing data in the genetic dataset were imputed based on the most frequent genotype in the sampling site and we build the model using the lfmm_ridge function of the R package *lfmm* (Caye et al., 2019). The number of latent factors (*K* value) and regularization parameters (lambda) were respectively set to 9 and 1e^−5^, to minimize predictor error estimated by a cross‐validation method, as advised in the *lfmm* manual. *p*‐Values were calibrated using the genomic control method, and false discovery rate (*q*‐value) was calculated following the Benjamini–Hochberg procedure in the *qvalue* R package (Storey et al., 2020). Associations between a SNP and environmental factor with *q*‐value < 0.01 were considered statistically supported.

## 3 RESULTS

Based on mtDNA, we only detected secondary contact between glacial lineages in Labrador: Samples from ANA and KAM corresponded to previously published haplotypes from either the Atlantic (*n* = 23; ATL1, ATL4) or Arctic (*n* = 11; ARC19, ARC22) lineage. Mitochondrial haplotypes from Nunavik and Baffin Island samples (*n* = 114) all corresponded to haplotypes from the Arctic lineage, primarily ARC19 (*n* = 85), but also ARC22, ARC25, and ARC26 (Table S1).

Using GBS, we sequenced a total of 745 samples, of which 95 did not pass our filtering criteria. Retained individuals (*n* = 650) had 2.217 million reads on average. A total of 30,773 SNPs were called and passed basic filtering in STACK2. Among these, 7244 SNPs were categorized as duplicates and removed (Figure S1) and 5009 SNPs were pruned during linkage disequilibrium assessment. We then identified and removed 408 SNPs linked to sex, 139 of which mapped on the sex chromosome (15.6% of SNPs on this chromosome) and 269 were elsewhere in the genome (1.5% of SNPs outside the sex chromosome). The final dataset used in subsequent analyses comprised 18,112 SNPs (see Table S2 for exact criteria and number of filtered SNPs), with a global 6.87% missing genotypes. Bayescan identified 273 SNPs as outliers (*q*‐value < 0.05), 170 of those had higher *F*_ST_ values than expected under neutrality, suggesting divergent selection, and 103 had lower *F*_ST_ values than expected, suggesting balancing selection. Pcadapt identified 186 outliers (*q*‐value < 0.05), including three SNPs commonly identified by both genome scan methods, for a total of 456 markers putatively under selection. The remaining 17,676 SNPs were considered neutral for subsequent analyses.

### 3.1 Population structure

**FIGURE 2:**
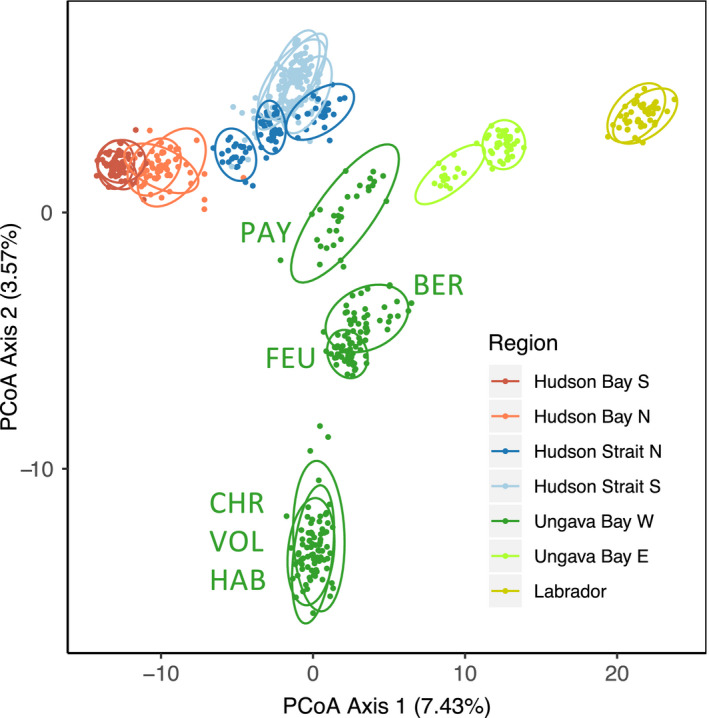
(a) Population structure assessed by a principal coordinate analysis (PCoA). Individual scores on PCoA axes 1 and 2 are presented as points and colored by a priori geographical region. An ellipse representing a 95% confidence interval was drawn around each sampling site. The percentage of genetic variance explained by each axis is in parentheses

**FIGURE 3:**
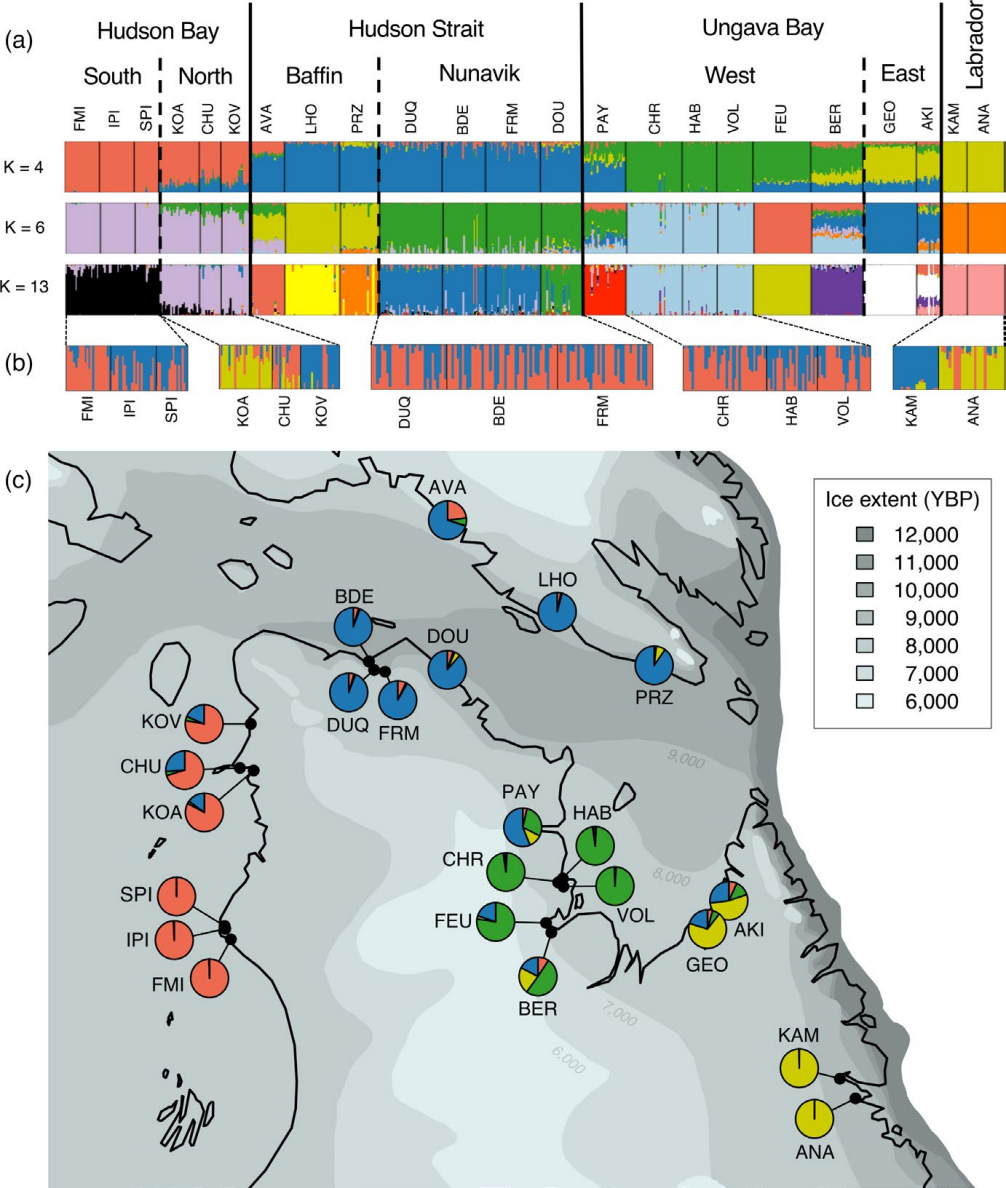
(a) Results of the hierarchical Bayesian clustering analysis implemented in ADMIXTURE for a number of genetic clusters (*K*) of 4, 6, and 13. (b) Lower rows display the results for separate analyses on sampling sites sharing similar membership to clusters at *K* = 13. (c) Results of ADMIXTURE for *K* = 4 clusters, with individual ancestry averaged by sampling site and represented by pie charts. Approximative extent of glaciers, adapted from the noncalibrated isochrones from Dalton et al. (2020), is represented by shades of gray for 6000–12,000 years before present (YBP)

Population statistics are presented in Table 2. N_e_ estimates ranged from 62 to 810 and were not correlated with log‐transformed catchment area (*r*_s_ = 0.18, *p* = 0.202). The first axis of the PCoA reflected the longitude of the sampling sites, while the second axis differentiated populations in Western Ungava Bay from all other sampling sites (Figure 2). Sampling sites within Hudson Bay, Hudson Strait, and Labrador are, respectively, overlapping on the first two PCoA axes. The number of genetic clusters best supported by an ADMIXTURE analysis with all sampling sites was 13 (Figure S2 for comparison of cross‐validation errors). At this level, individuals within a sampling site were generally homogenous in their membership to clusters (Figure 3a, see Figure S3 for *K* = 2–15). Multiple sampling locations sometimes clustered together, this was the case for the two sites in Labrador, two groups of three neighboring sampling sites in Northern and Southern Hudson Bay, and all pairs of rivers with a common estuary except the Leaf (FEU) and Bérard (BER) rivers. ADMIXTURE best supported *K* = 1 within each of those groups. Even if cross‐validation errors for *K* = 2 were in some cases only slightly higher (Table S3), individual cluster membership for *K* > 1 was then at most loosely linked to sampling sites (Figure 3b). However, Labrador sites, where samples were collected as juveniles, are a notable exception.

**TABLE 2:** Summary of Arctic Char sampling and associated descriptive statistics. Samples were collected on adults in rivers and lakes, with exceptions († in estuaries, ‡ on juveniles)

Region code	Name	LON	LAT	*n*	*H*_o_	*H*_e_	Polymorphic SNPs	N_e_ estimate (parametric CI 95%)
Nunavik								
Hudson Bay								
Southern								
IPI	Ipikituk River	−78.38	58.73	24	0.272	0.254	9748	194.8 (191.0–198.8)
SPI	Saputaliuk River	−78.36	58.70	17	0.292	0.260	9519	61.6 (61.0–62.3)
FMI	Five Mile Inlet	−78.21	58.56	24	0.250	0.258	10,060	114.5 (113.2–115.9)
Northern								
KOA	Korak River	−77.63	60.75	28	0.278	0.282	11,219	330.1 (322.4–338.2)
CHU	Chukotat River	−78.02	60.79	15	0.283	0.289	10,554	504.3 (469.4–544.9)
KOV	Kovik River	−77.70	61.36	21	0.267	0.282	10,704	77.6 (76.9–78.2)
Hudson Strait								
BDE^†^	Déception Bay	−74.62	62.13	30	0.317	0.320	12,403	321.1 (315.0–327.5)
DUQ	Duquet Lake	−74.53	62.06	44	0.313	0.318	12,759	490.7 (481.9–499.8)
FRM	François‐Malherbe Lake	−74.25	62.04	38	0.317	0.320	12,691	609.8 (593.9–626.6)
DOU	Douglas Harbour	−72.65	62.06	29	0.308	0.316	11,912	160.7 (159.0–162.4)
Ungava Bay								
Western								
PAY	Payne River	−70.70	60.01	30	0.300	0.311	11,791	98.9 (98.3–99.6)
CHR	Red Dog River	−69.79	59.30	39	0.291	0.294	11,500	171.5 (170.0–172.9)
VOL	Voltz River	−69.66	59.25	25	0.302	0.293	10,698	235.5 (230.9–240.2)
HAB^†^	Hopes Advance Bay	−69.63	59.32	24	0.296	0.298	10,864	214.6 (210.5–218.8)
FEU	Leaf River	−70.11	58.77	40	0.268	0.264	9681	809.6 (777.9–844.1)
BER	Bérard River	−69.97	58.65	36	0.311	0.320	12,406	93.8 (93.4–94.3)
Eastern								
GEO	George River	−65.95	58.69	37	0.313	0.314	12,036	786.7 (758.6–816.9)
AKI	Akilasaaluk River	−65.40	59.06	17	0.312	0.320	11,521	170.0 (166.5–173.6)
Baffin Island								
AVA	Ava's Inlet	−72.64	64.01	23	0.285	0.289	11,148	253.5 (247.5–259.8)
LHO	Lake Harbour	−69.82	62.82	38	0.313	0.291	11,330	295.3 (290.9–299.9)
PRZ	Pritzler Harbour	−67.32	62.12	27	0.299	0.303	11,821	125.3 (124.1–126.6)
Labrador								
KAM^‡^	Kamanatsuk River	−62.54	56.74	18	0.310	0.307	11,656	302.9 (292.8–313.6)
ANA^‡^	Anaktalik Bay	−62.15	56.49	26	0.302	0.300	12,011	80.7 (80.1–81.2)

**FIGURE 4:**
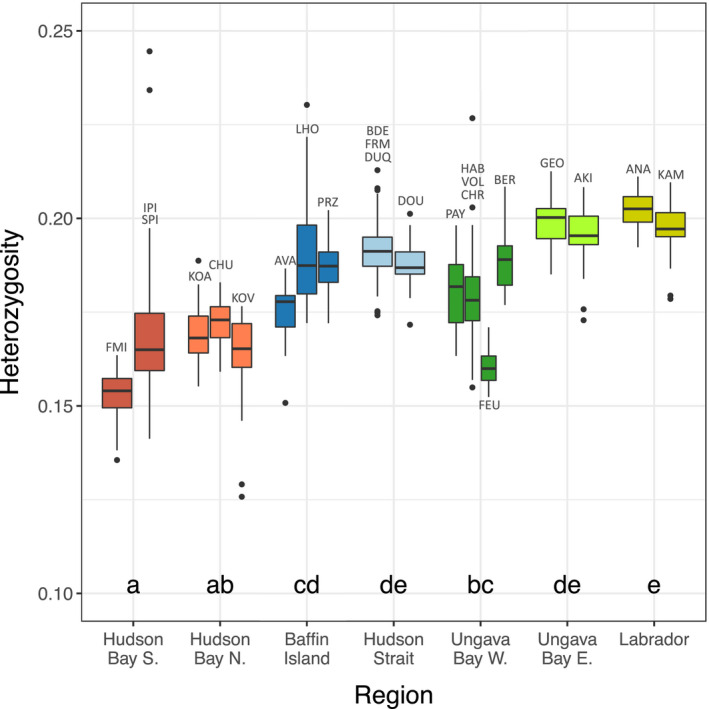
Individual proportion of heterozygous SNP markers at each sampling site, colored by region. Sampling sites on connecting rivers and estuaries were grouped, except for FEU and BER. For each boxplot, bold line indicates mean, the box limits 25th and 75th percentile, and whiskers represent 10th and 90th percentile. Letters indicate group membership of the regions based on a comparison of least‐square means in a mixed‐effect model (*p* < 0.05) with the individual locations as a random effect

Individual mean proportion of heterozygous markers varied regionally (Figure 4). Notably, southern Hudson Bay displayed the lowest observed proportion of heterozygous markers, and both southern and northern Hudson Bay had lower values than Hudson Strait (*p* < 0.05). In Ungava Bay, eastern sampling sites had a diversity similar to Labrador and higher diversity than western sampling sites.

### 3.2 Landscape genomics

**FIGURE 5:**
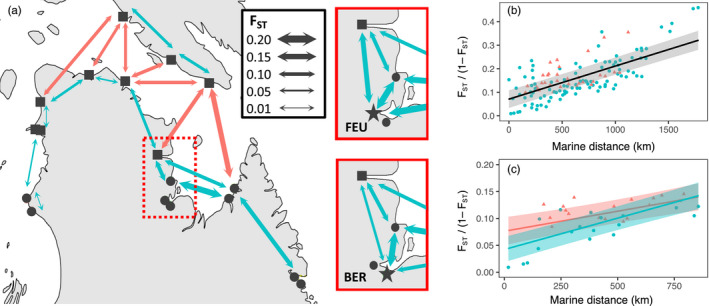
(a) Pairwise *F*_ST_ between neighboring sampling sites are shown, with line thickness proportional to values. Red arrows indicate pairs of sites on opposite sides of the Hudson Strait, while blue arrows link pairs on the same shore. All pairwise *F*_ST_ values are presented in Table S3. Red extent is magnified to contrast *F*_ST_ between Leaf (FEU) and Bérard (BER) rivers (pairwise *F*_ST_ = 0.135), represented by a star symbol in their respective subpanels. Other pairs of adjacent sampling sites had their *F*_ST_ values averaged for better visualization. (b, c) Isolation‐by‐distance is represented according to the relation between marine distances and linearized pairwise *F*_ST_, which is estimated between pairs of sampling sites separated by Hudson Strait (red triangles) and on the same coast (blue dots). Regression lines and 95% confidence intervals are plotted for mixed‐effect models with fixed effects including marine distance, crossing of Hudson Strait and the interaction of those factors. Models were fitted using (b) all sampling sites and (c) only sampling sites within 250 km of Hudson Strait (i.e., squares on panel a)

*F*_ST_ calculated by pairs of sampling sites ranged between 0.000 and 0.319 across neutral loci and between −0.003 and 0.544 across putatively adaptative loci (Figure 5a, Table S4). *F*_ST_ for adaptive loci were roughly twice the value for neutral loci and both measures were highly correlated (slope = 1.78, *r* = 0.94). All comparisons using neutral loci were statistically supported at *p* = 0.01, except for a pair of sampling sites on rivers sharing an estuary (*p*_CHR‐VOL_ = 0.568) and a pair of sampling sites on a river and its estuary (*p*_BDE‐FRM_ = 0.015). These results indicate that all unconnected systems harbor genetically distinct populations, although their extent of differentiation is highly variable.

When examining IBD over all sampling sites, a positive correlation was found between linearized *F*_ST_ and marine distances (Figure 5b), but the inclusion of the effect of crossing the Hudson Bay (CROSSHS) did not improve the model, based on marginal *R* ^2^ (Table 3). However, similar analyses focusing on sampling sites within 250 km of the Hudson Strait supported that the slope and intercept were different for pairs of populations on either side of the Hudson Strait and pairs on the same coast, suggesting that populations separated by the Hudson Strait are more differentiated (Figure 5c).

**TABLE 3:** Parameters of isolation‐by‐distance mixed‐effect models, displaying degrees of freedom (df), conditional Akaike information criteria (cAIC), and marginal R‐squared (*R* ^2^m) compared to the null model

	All sites			Sites within 250 km of Hudson Strait		
	df	cAIC	*R* ^2^m	df	cAIC	*R* ^2^m
FST ~ 0	17.18	−362.18	—	8.06	−144.37	—
FST ~ DIST	18.95	−553.38	0.655	10.62	−199.86	0.535
FST ~ DIST + CROSSHS	19.80	−553.02	0.651	11.65	−211.28	0.626
FST ~ DIST * CROSSHS	20.80	−552.02	0.649	12.64	−214.37	0.642

### 3.3 Gene–Environment Association

Environmental factors were summarized with three PC axes (Table 1). The first axis (PC1; 38.0% of variation) was associated with primary productivity, dissolved oxygen, precipitation, and winter air temperature. The second axis (PC2; 26.5%) was associated with summer temperature, both in the marine and in freshwater habitats, as well as to turbidity and salinity. Finally, the third axis (PC3; 17.2% of variation) related mostly to tidal amplitude and partly to the size of the catchment area (Figure S4, S5).

The distance‐based Moran's eigenvector map produced 12 eigenvectors reflecting negative autocorrelation and four eigenvectors for positive autocorrelation (MEM1—4, see Figure S6). Model selection for the RDAs excluded MEM3 (*p* = 0.13), MEM4 (*p* = 0.21). MEM1 was highly correlated with PC3 (*r* = 0.71) and MEM2 was linked to PC1 (*r* = 0.74). We conducted a redundancy analysis (hereafter referred to as “RDA”) which excluded the spatial covariables MEM1 and MEM2 (thereby excluding factors correlated to the environment). A partial RDA (“pRDA”) that included the spatial covariables MEM1 and MEM2 was also conducted. Spatial covariables explained 34.8% of the genetic variance, 29.0% of which was shared with environmental factors, resulting in the RDA and pRDA having respective adjusted *R* ^2^ of 0.438 and 0.148.

**FIGURE 6:**
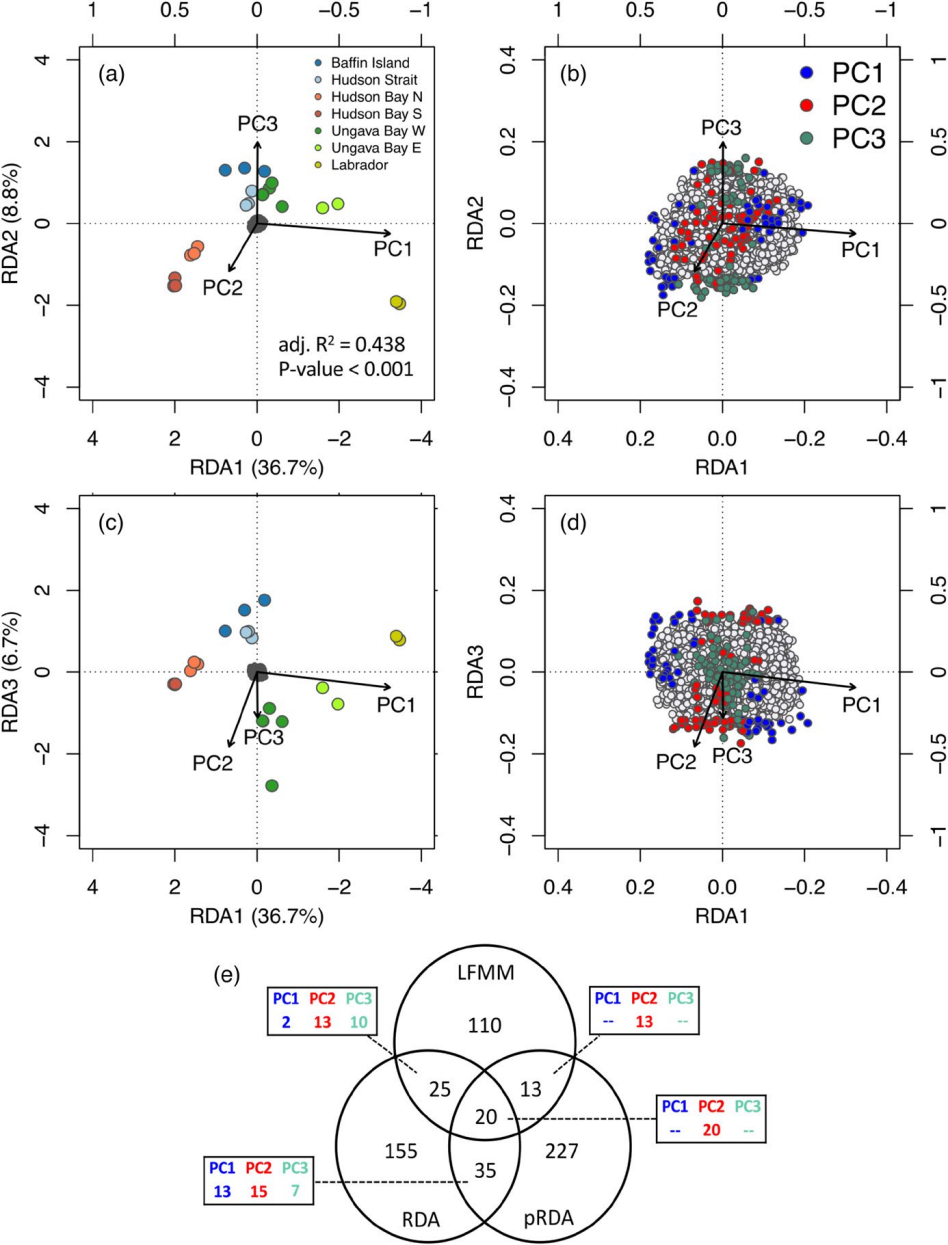
Triplots for (a) axes 1–2 and (b) axes 1–3 in a RDA excluding spatial components. The dark gray cloud of points at the center of each plot represents the SNPs and colored points represent sampling sites with color coding by region. Triplots are magnified to highlight SNP loadings on (c) axes 1–2 and (d) axes 1–3. Candidate SNPs are shown as colored points with coding by most correlated environmental predictor (see Methods for description of predictors). Vectors represent environmental predictors according to the scales on top and right axes. (e) Intersection of candidate SNPs detected in a latent factor mixed model (LFFM) and redundancy analyses with (pRDA) and without (RDA) spatial correction. For each intersection, the box indicates the distribution of environmental components associated with candidate SNPs

In both the RDA and the pRDA, the first three axes explained significant portions of the variance (*p* < 0.05) and were thus used for outlier detection. The RDA identified only 14 outliers on the first axis and respectively 112 and 109 on the second and third axes (Figure S7), for a total of 234 markers on 38 *Salvelinus sp.* linkage groups (Figure S8). The environmental components PC1, PC2, and PC3 had, respectively, 62, 83, and 90 of those outliers most correlated to them. The pRDA identified a total of 295 outlier SNP markers distributed across 36 linkage groups (Figure S9). Respectively 98, 113, and 84 outliers were found on each of these three axes (Figure 6). Those outliers were generally most correlated to PC1 (*n* = 185) or PC2 (*n* = 90). LFMM identified a total of 173 outlier SNPs (q < 0.01, Figure S10), 13 of which being associated PC1, 102 associated with PC2, and 58 to PC3. A total of six SNPs were significantly associated with more than one environmental component, five of which were associated with both PC2 and PC3.

Of the 585 unique outliers, 57 (9.7%) were also among the loci considered as potentially under selection by either genome scan methods (i.e., pcadapt and Bayescan). This relatively low proportion is not unexpected, given that genome scans are mostly sensitive to large‐effect loci, while RDAs have the power to detect polygenic selection (Forester et al., 2018). A total of 93 (15.9%) outliers were identified as associated with an environmental factor by at least two GEA methods (Figure 6e, see Figure S11 for spatial distribution of allele frequencies and Table S6 for the number of outliers per linkage group). Among 15 of those 93 candidate SNPs, the LFFM identified PC2 as the environmental factor associated with the candidate SNPs while the factor most correlated to genotypes in the RDA/pRDA was PC1. In those cases, we reported PC2 as it was also statistically correlated to genotypes (*r* > 0.45, *p* < 0.05). Thus, 15 candidate SNPs were associated with PC1 (precipitation, winter air temperature, and productivity), 61 were associated with PC2 (air and sea‐surface summer temperature, salinity, and turbidity), and 17 were associated with PC3 (tides). We identified a total of 75 named genes within 10,000 bp of those candidate SNPs, covering a wide range of biological functions, including muscle contraction, development, and circadian rhythm (Table S7).

## 4 DISCUSSION

Given their homing behavior, anadromous salmonids are considered prime models for understanding local adaptation (Fraser et al., 2011; Hendry & Stearns, 2004) and landscape genomics studies have provided key information for the conservation and management of economically important species (Waples et al., 2020). Here, we documented patterns of neutral and adaptive genetic variation in anadromous Arctic Char populations in the Nunavik region using genomic data. By combining fine‐ and broad‐scale sampling, our results revealed a hierarchical genetic structure whereby neighboring hydrographic systems generally harbored distinct populations that could be grouped within oceanographic basins, while differentiation was also influenced by isolation‐by‐distance. To assess the potential for local adaptation to the contrasted environments in our vast study area, we combined three GEA methods. By doing so, we found genomic signatures that were consistent with local adaptation to either or both freshwater and marine habitats.

### 4.1 Neutral structure in a postglacial context

By 8000 YBP, most of Hudson Strait and the coast of Labrador was free of ice, while the coasts of Hudson Bay and Ungava Bay deglaciated around 6500‐7200 YBP (Dalton et al., 2020). The decreasing genetic diversity we observed along the coast from Hudson Strait to southern Hudson Bay, as well as low *F*_ST_ values between populations within the Hudson Bay region, is therefore consistent with expectations following a recent range expansion (Eckert et al., 2008; Goodsman et al., 2014). Similar patterns were found in the Coho Salmon (*Oncorhynchus kitshush*) (Rougemont et al., 2020), in European lamprey species (*Lampetra* spp.) with diversity and differentiation decreasing in populations far from the Iberic glacial refugia (Mateus et al., 2016), and in Scottish populations of Atlantic Salmon, where genetic diversity was lower in more recently deglaciated regions (Cauwelier et al., 2018). Following the initial recolonization of rivers in Nunavik by Arctic Char, processes such as isostatic rebound and the formation of proglacial lakes considerably modified the drainage basins (Dubé‐Loubert et al., 2018; Jansson, 2003), which could have contributed to gene flow between regions (e.g., Ruzzante et al., 2020).

In Northern Labrador, Salisbury et al. (2019) found extensive admixture of the Arctic mitochondrial lineage, which crossed the Canadian Arctic Archipelago from a glacial refugium in the western Arctic (Brunner et al., 2001; Moore et al., 2015), and the Atlantic lineage, which we expect to have crossed the Atlantic Ocean from the Palearctic during the last deglaciation (Brunner et al., 2001; Wilson et al., 1996). Our results were consistent with these previous studies as we did not observe any Atlantic lineage haplotypes in populations outside Labrador. However, similar to Moore et al. (2015), we found evidence for introgression from the Atlantic lineage in our nuclear data from southern Baffin Island, and even stronger evidence in Eastern Ungava Bay, where our ADMIXTURE results showed shared ancestry with Labrador samples. Such mito‐nuclear discordance is not uncommon in cases of adaptive introgression and could also be linked to sex‐biased dispersion (Toews & Brelsford, 2012) as it is suggested that Arctic Char males are more mobile than females (Dempson & Kristofferson, 1987; Moore et al., 2016). Nevertheless, admixture in the eastern part of our study area could explain the higher genetic diversity, as expected during secondary contact of marine fishes (Bay & Caley, 2011; Grant & Bowen, 1998).

### 4.2 Contemporary gene flow

Our results revealed a hierarchical genetic structure with most geographic regions comprising distinct populations of Arctic Char in every sampled river not sharing an estuary. However, population structure in Hudson Bay was weaker, as there were signs of admixture between rivers within 100 km of each other. Other studies have attributed low genetic differentiation over long distances to recent postglaciation colonization events (Delgado et al., 2019; Moore et al., 2013; O’Malley et al., 2019). Nonexclusively, lower salinity and a longer summer period in Hudson Bay could also lead to higher connectivity between estuaries.

Pairs of populations on opposite shores of the Hudson Strait did not deviate from IBD, which, contrary to our expectation, suggests ongoing gene flow over long marine distances. In contrast, we did find limited evidence for Hudson Strait acting as a barrier to gene flow at a finer spatial scale, that is, by restricting analyses to sampling sites around the strait. Differentiation between populations separated by the Hudson Strait was lower than, for example, pairwise *F*_ST_ between populations in western and eastern Ungava Bay, despite those populations being connected by a coastal migratory route. We cannot exclude the possibility of other barriers to gene flow in our study area, as near‐shore oceanographic features could also limit dispersal (e.g., Quéméré et al., 2016), but differentiation in Ungava Bay could also be partly driven by nuclear introgression from the Atlantic lineage in the eastern sampling sites. Additionally, populations on either side of Hudson Strait could share ancestral polymorphism rather than exhibit contemporary gene flow. These other processes (introgression and ancestral shared polymorphism) could hide the effect of the Hudson Strait as a potential barrier to gene flow. As such, our results hint again at the relative importance of postglacial recolonization over contemporary restrictions to gene flow on population structure in this recently deglaciated area.

Arctic Char is known for its higher straying rates than most other salmonids, but many studies argue that this dispersal does not necessarily lead to gene flow, as individuals are more prone to straying when overwintering than when spawning (Dempson & Kristofferson, 1987; Moore et al., 2013; Moore et al., 2017; Sévigny et al., 2020). In this study, spawning and nonspawning individuals were not distinguished upon sampling, which might have resulted in underestimating the real genetic differentiation among rivers. Sites sharing an estuary generally did not exhibit population structure, indeed suggesting high levels of straying between nearby rivers, but our ADMIXTURE results indicate that straying is very rare at the inter‐regional scale this study mostly focusses on. Surprisingly, however, the Leaf River (FEU) and the Bérard River (BER) showed strong evidence of genetic differentiation despite sharing an estuary. Interestingly, those two rivers are found on either side of the limit of two major geological provinces (Thériaut & Beauséjour, 2012). As salmonids rely partly on their olfaction to recognize their natal rivers (Keefer & Caudill, 2014), the distinct geologies of the Leaf and Bérard systems could perhaps improve their homing ability and thus limit straying in these systems. Finally, the two sites in Labrador also displayed clearer signs of differentiation than other pairs of neighboring rivers, perhaps because the sampled juveniles did not have the opportunity to stray yet. On the other hand, gene flow between Labrador populations might also be lower than in other regions of the Canadian Arctic, as Li et al. (2020) found population structure in the Lower Northwest Passage, Nunavut, to be similar to what we measured in Nunavik.

### 4.3 Genomic evidence consistent with local adaptation

We explored how genetic variation in Arctic Char was linked to a range of climatic and physical environmental predictors while considering both freshwater and marine habitats, something that has rarely been done in salmonids (but see Bekkevold et al., 2020). While different GEA methods identified candidate SNPs with every environmental component considered, we found that most of the best‐supported candidates were associated with the component reflecting summer SST and air temperature, salinity, and turbidity (PC2).

In our study, we treated the environment experienced by anadromous Arctic Char over their life cycle as a set of collinear variables, hence leading to our inability to differentiate signatures of selection in the freshwater and marine habitats. We also created two sets of habitat‐specific environmental components, but marine and freshwater components were strongly correlated and created several instances of candidate SNPs associated with both a marine and freshwater component by GEA methods (results not shown). Nevertheless, using those habitat‐specific components, we did find through a constrained ordination that both habitats independently explained significant proportions of the genetic variation. For instance, measures of air temperature (as a surrogate for river temperatures) and sea temperatures were highly correlated, but likely caused selective pressures at different life stages. Several studies have found temperature to be a driver of genetic structure and local adaptation in salmonids, with most studies focusing on freshwater measures (Bourret et al., 2013; Dionne et al., 2008; Perrier et al., 2017; Sylvester et al., 2018). In contrast, selective pressures in marine habitats have been argued to be weaker since observed mortality rates are lower at sea (Garcia de Leaniz et al., 2007; Quinn, 2005). However, SST near the mouth of spawning river was found to be correlated with timing of migration in species of Pacific salmon (Kovach et al., 2015) and to phenology‐related genes in Arctic Char (Madsen et al., 2019). Sampling sites across our study area displayed considerably different SST conditions: While coastal waters surrounding sampling sites in Hudson Bay reached mean summer SSTs ranging from 5.5 to 7.5°C, Hudson Strait stayed closer to the freezing point (0.5–2°C). Such contrasts in surface temperature, coupled with a discrepancy in tidal regimes and salinity, likely produce widely different coastal habitats, which we argue could result in local adaptation of Arctic Char populations.

Recent studies of local adaptation in salmonids have focused on tributary‐specific variation in freshwater conditions within a single catchment (e.g., the Columbia River; Hand et al., 2016; Hecht et al., 2015; Micheletti et al., 2018). However, the freshwater environmental factors used in our study are catchment‐based given our sampling strategy, which prevents us from knowing the precise spawning site or overwintering lake. In marine systems, genomic evidence for local adaptation and isolation‐by‐environment has been found both at local scales in heterogeneous habitats (e.g., Lehnert et al., 2019; Miller et al., 2019) and over large geographic distances (e.g., Clucas et al., 2019). Arctic Char is expected to use preferred habitats based on temperature, salinity, and prey availability (Harris et al., 2020; Spares et al., 2012, 2015). As we averaged near‐shore marine conditions around river mouths, this study is limited to broad‐scale environmental heterogeneity. This is in line with the suggestion by Fraser et al. (2011) that adaptation of anadromous salmonids to the marine environment should occur at a larger spatial scale than in fresh water.

Regardless of the geographic scale being studied, an ideal sampling design for detecting local adaptation should maximize the environmental variation while minimizing its collinearity with neutral genetic patterns. For example, Lotterhos and Whitlock (2015) suggested sampling pairs of populations with similar ancestry and contrasting environments. Such a design is suitable when studying variables that may change drastically over short distances, such as catchment area and upstream migration distances. However, climatic and physicochemical conditions experienced by geographically close populations of Arctic Char are more likely to be similar than in distant ones. Similar considerations were discussed in Nadeau et al. (2016), where patterns of IBD, isolation‐by‐environment, and isolation‐by‐colonization were difficult to disentangle for two pine species in a recently recolonized range. In our study, some genotypes for GEA candidates (Figure S12) have varying allele frequencies that follow spatial patterns reminiscent of the neutral structure we described earlier, especially in PC1, where environmental variation followed a longitudinal gradient. In some of those cases, it is possible that a GEA was detected even though neutral processes could better explain the distribution of the observed allele frequencies. For example, if colonization of Hudson Bay did occur by rapid demographic expansion as discussed earlier, allele‐surfing events, where a mutation can reach high frequency by chance alone (Edmonds et al., 2004), could explain differential allele frequencies (Rougemont et al., 2020). Alternatively, the introgression of the Atlantic glacial lineage in the eastern part of the study area could also have led to divergence due to drift during the LGM to be falsely identified as linked to environmental variation. On that note, the pRDA and LFMM methods all accounted in some way for neutral or spatial structure. However, it is noteworthy that even the RDA, while excluding spatial correction, only identified a few outliers whose variation was driven by the Labrador sampling sites, that is, from the Atlantic glacial lineage. As we found a significant part of genetic variation to be explained jointly by environmental variation and spatial patterns, we advocate that including GEA methods that do not account for spatial structure offer a way to acknowledge that local adaptation can also contribute to genetic structure across contrasting environments, for instance by selecting against maladapted migrants (Dionne et al., 2008; Wang & Bardburd, 2014). In that sense, local adaptation in the system studied here could reinforce the neutral structure discussed earlier.

### 4.4 Implications for conservation and management

Genomic data have the potential to improve the definition of management and conservation units by accounting for neutral genetic structure as well as local adaptation (Bernatchez et al., 2017; Funk et al., 2012). Arctic Char populations in Nunavik support important small‐scale subsistence fisheries, and stocks are managed on a river‐by‐river basis on the premise that each river contains a single and distinct population (Johnson, 1980). However, the weak genetic differentiation in rivers sharing an estuary and our inability to identify substructure at this level suggest frequent straying of adults, in line with the evidence for the prevalence of mixed stocks of Arctic Char in adjacent rivers (Boguski et al., 2016; Moore et al., 2014, 2017). If genomic tools were to be used for stock assignment of adult fish (e.g., Meek et al., 2016; Moore et al., 2017), further local sampling including juveniles and/or reproducing adults would be useful to better understand fine‐scale population structure. The evidence we found for local adaptation to environments is in concordance with the neutral genetic structure at a broader scale, as major oceanographic basins around Nunavik are contrasted both in their environments and in the ancestry of their Arctic Char populations. We therefore suggest that these regional differences between Hudson Bay, Hudson Strait, and Ungava Bay, with distinction of the western and eastern coast for the latter, should form the basis of management actions at a regional level.

Also, there is growing interest in Arctic Char hatchery projects in Nunavik, both for supplementation and for reintroduction of Arctic Char in traditional fishing locations (George, 2007; Rogers, 2015). The genetic information gathered here could be useful for this purpose, and the adaptative variation explored in this study highlights the need for careful selection of source populations for brood stocks, as maladapted domesticated individuals can waste efforts and resources, in addition of likely being detrimental to wild populations (Fraser et al., 2010; Tymchuk et al., 2006).

As the Arctic warms at a greater pace than any other regions on earth (Cohen et al., 2014), there might be concerns about the response of Arctic Char populations to their changing environment. Traits that are currently optimally adaptive in the present environment could eventually become maladaptive. Species will thus likely need to shift their distributions poleward and/or will need to adapt to persist in their current distribution if there is presence of appropriate genetic diversity/phenotypic plasticity. Layton et al. (2021) highlighted the genomic vulnerability to the future climate in Arctic Char from Labrador, especially at lower latitudes where higher temperatures are expected to be observed. A temporal study recently showed that Arctic Char populations in Greenland have exhibited stable genetic structure over the last 60 years in face of rapid climate change, and argued that gene flow, although low, could allow for a modest level of evolutionary rescue in the short term (Christensen et al., 2018). Our study shows potential for local adaptation of Arctic Char populations to both their marine and freshwater habitats. As changes in climate might operate at a different pace, scale, and stability in marine and terrestrial ecosystems (Burrows et al., 2011), there is a need for continued research about the interaction of selective pressures over the lifespan of anadromous organisms.

## CONFLICTS OF INTEREST

No conflict of interest has been declared by the authors.

## Supporting information

Figures S1‐S11

Tables S1–S7

## Data Availability

Raw sequences that support the findings of this study are openly available on NCBI SRA, accession number PRJNA655216 (https://www.ncbi.nlm.nih.gov/bioproject/PRJNA655216/).
